# *Toxoplasma gondii* Chitinase Induces Macrophage Activation

**DOI:** 10.1371/journal.pone.0144507

**Published:** 2015-12-10

**Authors:** Fausto Almeida, Aline Sardinha-Silva, Thiago Aparecido da Silva, André Moreira Pessoni, Camila Figueiredo Pinzan, Ana Claudia Paiva Alegre-Maller, Nerry Tatiana Cecílio, Nilmar Silvio Moretti, André Ricardo Lima Damásio, Wellington Ramos Pedersoli, José Roberto Mineo, Roberto Nascimento Silva, Maria Cristina Roque-Barreira

**Affiliations:** 1 Departamento de Biologia Celular e Molecular e Bioagentes Patogênicos, Faculdade de Medicina de Ribeirão Preto, Universidade de São Paulo, Av. Bandeirantes 3900, Ribeirão Preto, SP, 14049–900, Brasil; 2 Departamento de Microbiologia, Imunologia e Parasitologia, Universidade Federal de Sao Paulo, São Paulo, SP, Brasil; 3 Departamento de Bioquímica e Biologia Tecidual, Instituto de Biologia, Universidade de Campinas, Campinas, SP, Brasil; 4 Departamento de Bioquímica e Imunologia, Faculdade de Medicina de Ribeirão Preto, Universidade de São Paulo, Av. Bandeirantes 3900, Ribeirão Preto, SP, 14040–900, Brasil; 5 Laboratorio de Imunoparasitologia, Departamento de Imunologia, Microbiologia e Parasitologia, Universidade Federal de Uberlândia, Av. Pará, 1720, Uberlândia, MG, 38400 902, Brasil; National University of Ireland—Galway, IRELAND

## Abstract

*Toxoplasma gondii* is an obligate intracellular protozoan parasite found worldwide that is able to chronically infect almost all vertebrate species, especially birds and mammalians. Chitinases are essential to various biological processes, and some pathogens rely on chitinases for successful parasitization. Here, we purified and characterized a chitinase from *T*. *gondii*. The enzyme, provisionally named Tg_chitinase, has a molecular mass of 13.7 kDa and exhibits a *Km* of 0.34 mM and a *Vmax* of 2.64. The optimal environmental conditions for enzymatic function were at pH 4.0 and 50°C. Tg_chitinase was immunolocalized in the cytoplasm of highly virulent *T*. *gondii* RH strain tachyzoites, mainly at the apical extremity. Tg_chitinase induced macrophage activation as manifested by the production of high levels of pro-inflammatory cytokines, a pathogenic hallmark of *T*. *gondii* infection. In conclusion, to our knowledge, we describe for the first time a chitinase of *T*. *gondii* tachyzoites and provide evidence that this enzyme might influence the pathogenesis of *T*. *gondii* infection.

## Introduction

Toxoplasmosis is a worldwide infectious disease caused by *Toxoplasma gondii*, an obligate intracellular apicomplexan parasite [1–3]. This disease constitutes an economic and public health problem in different countries. *Toxoplasma gondii* infects nearly one-third of the world’s population, but its prevalence varies from 10 to 80% depending on the economic, cultural, and health status of the region. In endemic countries, such as Brazil and France, 50–80% of the population is infected by the parasite [4]. Infection of most healthy individuals is asymptomatic, but immunocompromised individuals, especially those with acquired immunodeficiency syndrome (AIDS), can develop toxoplasmosis. Infection of developing fetuses can also occur and is life threatening [5–9].

Chitin is a homopolymeric form of β(1–4)-linked *N*-acetyl-D-glucosamine (GlcNAc) residues, which constitutes most of the exoskeleton of nematodes, arthropods, mollusks, and the cell wall of fungi (reviewed by [10]). Chitin is hydrolyzed by chitinases, which exert important functions in many organisms [11]. A number of protozoan parasites use chitin and chitinases in their life cycles. *Trypanosoma cruzi*, *Leishmania donovani*, and *Plasmodium* spp. do not produce chitin, but secrete chitinases that act during their life cycle in the invertebrate host. Notably, *Plasmodium* ookinetes initiate the invasion of the mosquito midgut by secreting a chitinase necessary to cross the chitin-containing peritrophic matrix en route to the epithelial cell surface [12–14]. This chitinase is crucial to the *Plasmodium* life cycle; silencing of this chitinase gene resulted in parasites that were impaired in their ability to form oocysts in the midgut of *Anopheles* mosquitoes. For this reason, this chitinase in *P*. *falciparum* became an immunological target for blocking malaria parasite transmission in humans. There is evidence that each protozoan parasite has evolved to use chitin and chitinases differently and in a stage-specific manner.

In *T*. *gondii*, chitin was first discovered during the tachyzoite to bradyzoite life-stage transition. During cyst formation, chitin became detectable in the cyst wall, as indicated by wheat germ agglutinin (WGA) binding [15], also reported for *Entamoeba* [16] and *Giardia* [17]. The composition of the WGA-binding material was confirmed by treating cysts with an exogenous chitinase, rendering the cysts unable to bind WGA, resulting in disruption of the cyst wall and bradyzoite release [15]. The detection of chitin also demonstrated that the parasitophorous vacuole is modified into the cyst wall, an event that occurs early in *T*. *gondii* differentiation [18]. More recently, Nance and collaborators [19] reported the chitinase-dependent control of *T*. *gondii* cysts in the mouse brain. The implicated enzyme is produced by a macrophage population in the brain and is activated in response to chitin within the cyst wall [19, 20]. This active mammalian chitinase (AMCase) accounts for the efficient degradation and destruction of chitin in the cyst wall, culminating in protozoan cyst clearance from the brain and increased host survival [19]. AMCase-deficient mice displayed impeded parasite eradication and lower survival rates when infected with *T*. *gondii* [19].

No chitinases are currently known to occur in *T*. *gondii*. Herein, we describe the purification and characterization of an active chitinase, *N*-acetyl-β-d-glucosaminidase (NAGase), from a virulent strain of *T*. *gondii* tachyzoites. We show that this enzyme induces macrophage activation, as manifested by pro-inflammatory cytokine production such as IL-12, TNF-α, and IL-6. Since these cytokines are associated with a protective immune response to *T*. *gondii*, we postulate that *T*. *gondii* chitinase might influence the pathogenesis of toxoplasmosis.

## Results

### Chitinase activity of *T*. *gondii* tachyzoites

**Fig 1:**
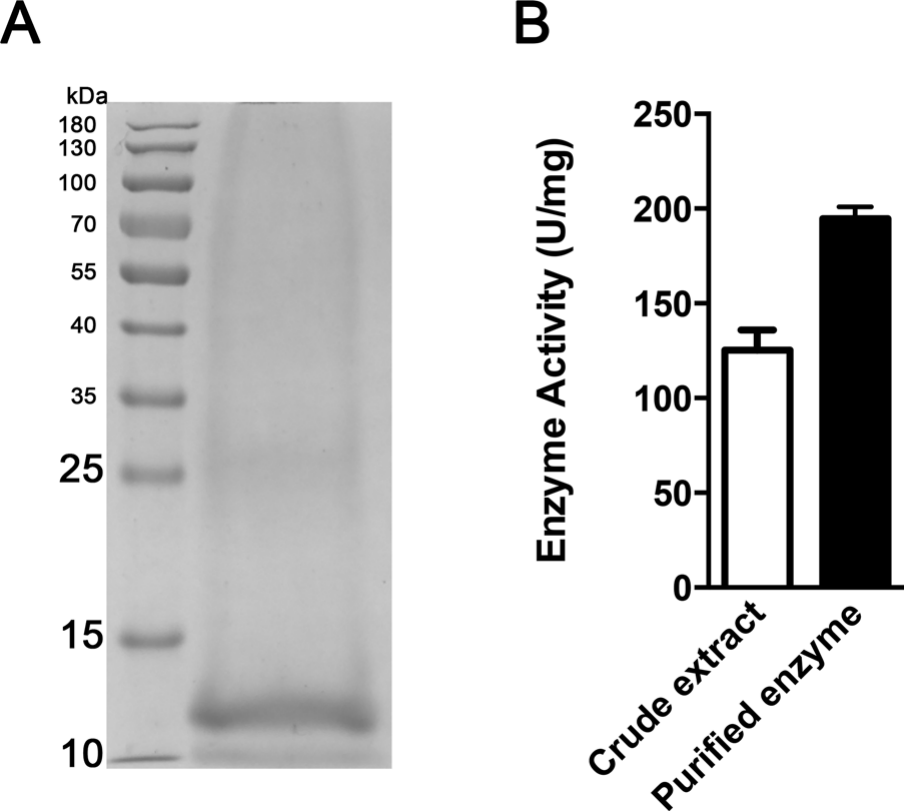
Chitinase activity from *T*. *gondii*. Soluble *T*. *gondii* antigens were purified by affinity with an IgY-Sepharose 4B column, and the chitinase activity of the delayed fractions was measured. (A) Electrophoresis analysis of purified chitinase from *T*. *gondii*. First lane—MW (Molecular Weight Ladder), second lane—purified chitinase in 12% SDS-PAGE. (B) Crude extract and purified chitinase activity from *T*. *gondii* was measured. Error bars represent standard errors calculated from three replicates.

We previously identified chitinase activity in *Paracoccidioides brasiliensis* extracts [21] and developed tools, such as specific antibodies, to characterize this enzyme (Pb_chitinase). These antibodies were also used to isolate chitinases from other pathogenic organisms [22]. *Toxoplasma gondii* tachyzoites (RH strain type I) were immobilized by anti-Pb_chitinase IgY on agarose beads and adsorbed a single protein from the total extract of the parasite. The molecular mass of the isolated component was 13.7 kDa, as determined by SDS-PAGE analysis (Fig 1A). The preparation displayed considerable NAGase activity, as verified by the release of *p*-nitrophenol from the substrate *p*-nitrophenol-β-*N*-acetyl glucosamine (pNPGlcNAc; Fig 1B). We assumed that the putative 13 kDa protein was responsible for NAGase activity, and we provisionally named the preparation Tg_chitinase.

**Fig 2:**
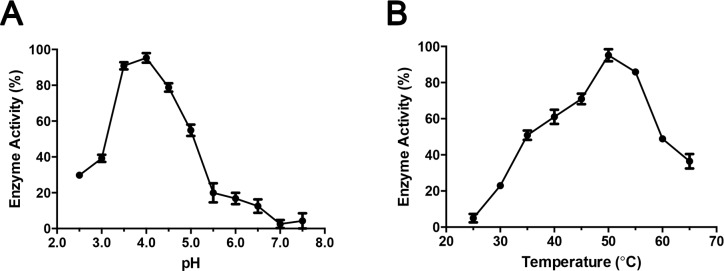
Effect of pH and temperature on *T*. *gondii* chitinase activity. Optimal (A) pH and (B) temperature profiles for Tg_chitinase from *T*. *gondii*. Maximum activity was standardized as 100% and enzymatic activities were measured at 405 nm. Data are representative of three independent assays.

To analyze the effects of pH and temperature on enzymatic activity, we evaluated Tg_chitinase over a 25–65°C temperature range (in 0.1 M phosphate citrate buffer at pH 4.0), and a 2.5–7.5 pH range. The optimal temperature and pH of the Tg_chitinase was estimated at 50°C and 4.0, respectively (Fig 2A and 2B). In addition, Tg_chitinase had a *Km* of 0.34 mM and a *Vmax* of 2.64 U/mg protein (Table 1).

**Table 1:** General catalytic properties of Tg_chitinase and other microbial chitinases.

Strain	Molecular mass (kDa)	pH	Temperature (°C)	Km (mM)	Vmax (U/mg)	Ref.
***Toxoplasma gondii***	**14**	**4.0**	**50**	**0.34**	**2.64**	This study
*Paracoccidioides brasiliensis*	70	5.0	37	0.28	3.25	[21]
*Trypanosoma cruzi*	48	5.5	-	1.5	-	[30]
*Pseudomonas fluorescens*	50	8.0	37	0.13	-	[45]
*Clostridium paraputrificum*	45.5	7.0	50	7.9	21.8	[46]
*Trichoderma harzianum*	36	4.0	50–60	8.06	3.36	[47]
*Streptomyces cerradoensis*	58.9	5.5	50	0.13	1.95	[48]

### Amino acid sequence and predicted 3D structure

**Fig 3:**
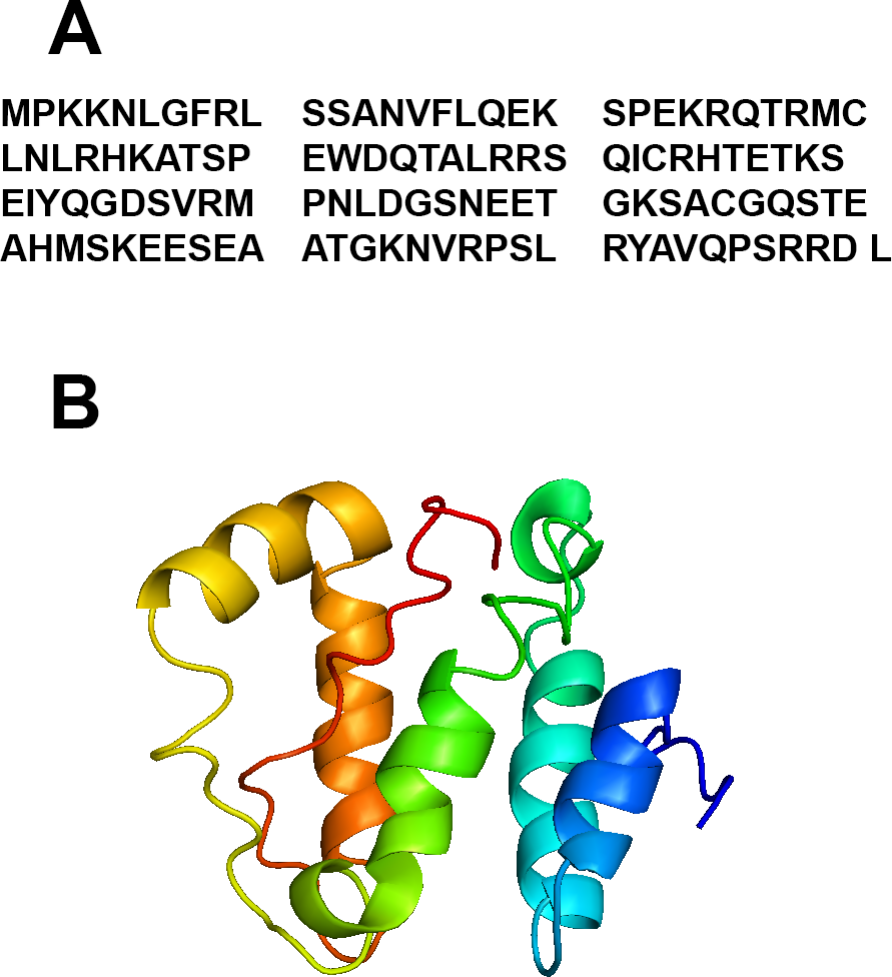
Amino acid sequence and predicted 3D structure of Tg_chitinase from *T*. *gondii*. Tg_chitinase from *T*. *gondii* was identified by mass spectrometry using the amino acid sequences of tryptic peptides listed in Table 2 (indicated in bold). (A) The amino acid sequence obtained from analysis of the peptide (SEIYQGDSVR) identified by BLASTP *T*. *gondii* GT1 (TGGT1_286465). (B) Predicted 3D structure of Tg_chitinase using I-TASSER.

The 13.7 kDa band, corresponding to the putative Tg_chitinase, was excised from the SDS-PAGE gel and subjected to *in situ* trypsin digestion followed by mass spectrometry. The minimum criterion for peptide matching was performed using Peptide Prophet [23] at a score greater than 0.8. Peptides that matched this criterion were further grouped by protein sequence using the Protein Prophet [24] algorithm and only proteins with an error rate of 5% or less were considered. Two peptide sequences were identified, one for a hypothetical protein (TGME49_286465; 13.6 kDa) and one for a peroxiredoxin PX3 (TGME49_230410; 30 kDa) (Table 2). The TGE49_286465 sequence was used to predict its 3D structure using I-TASSER [25] (Fig 3).

**Table 2:** LC-MS/MS mass spectrometry analysis of peptides generated by trypsin digestion of Tg_chitinase from *T*. *gondii*.

Ion Percent	Mr (calculated)	Delta	Peptide sequence
39%	1153.5480	-0.2780	SEIYQGDSVR
57%	966.5250	+0.4660	VTLSEVYR

The predicted protein did not show domains or binding sites following a BLAST search against the Pfam protein domain database [26]. Analysis using Phyre2 [27] showed 90% model confidence composed of 50% alpha helices and 13% beta strands. The model template was selected for alignment with other protein databases by using a heuristic method to maximize confidence. About 103 residues were modeled and showed low confidence (12%) to a transcriptional factor with an AT-rich interactive domain-containing protein 4a (40% similarity) and to a NagB/RpiA/CoA transferase-like protein (47% similarity). Notably, an analysis using the InterPro database revealed homology with a putative sugar-binding domain (IPR007324) of *Bacillus subtilis* [28], a result typical for chitinases. However, more structural studies are necessary to elucidate the real identity of this protein.

### *T*. *gondii* chitinase is located in the cytoplasm of the apical region of tachyzoites

**Fig 4:**
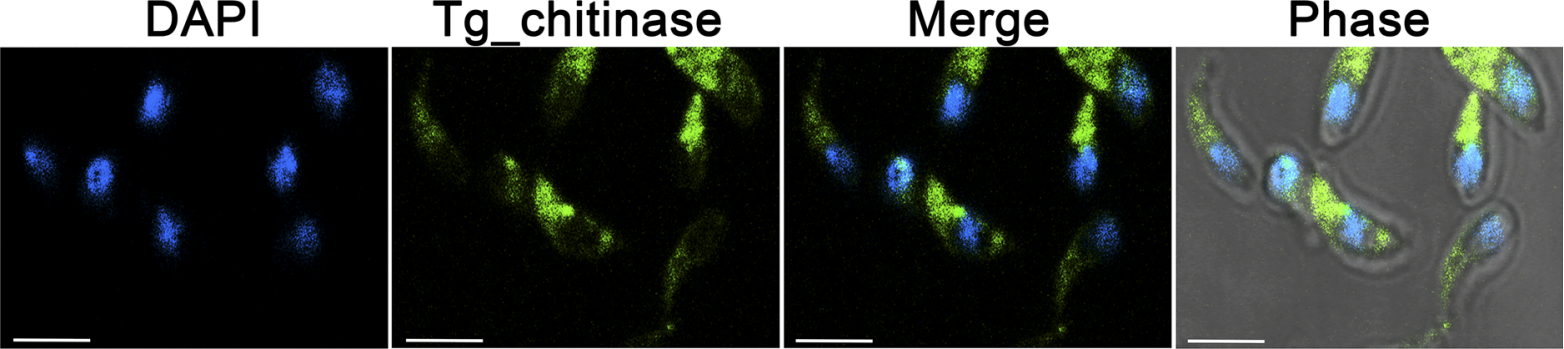
Tg_chitinase subcellular localization. (A) *T*. *gondii* tachyzoite DNA was stained with DAPI (blue), and (B) immunostained for Tg_chitinase with an anti-chitinase antibody, which was biotinylated with an antibody conjugated to anti-streptavidin-FITC (green). Superimposition of images stained for Tg_chitinase and DAPI (Merge and phase microscopy, C and D, respectively). Tg_chitinase is localized throughout the cytosol of *T*. *gondii* tachyzoites. Scale bar = 2.5 μm.

We determined the subcellular localization of Tg_chitinase by reacting tachyzoites (*T*. *gondii* RH strain) with IgY specific to the NAGase of *P*. *brasiliensis*. We determined that Tg_chitinase has a punctate distribution in the tachyzoite cytoplasm, being clearly prominent at the parasite apical extremity (Fig 4). This localization pattern suggests that Tg_chitinase may be actively involved in the invasion process of the parasite.

### *T*. *gondii* chitinase induces macrophage activation

**Fig 5:**
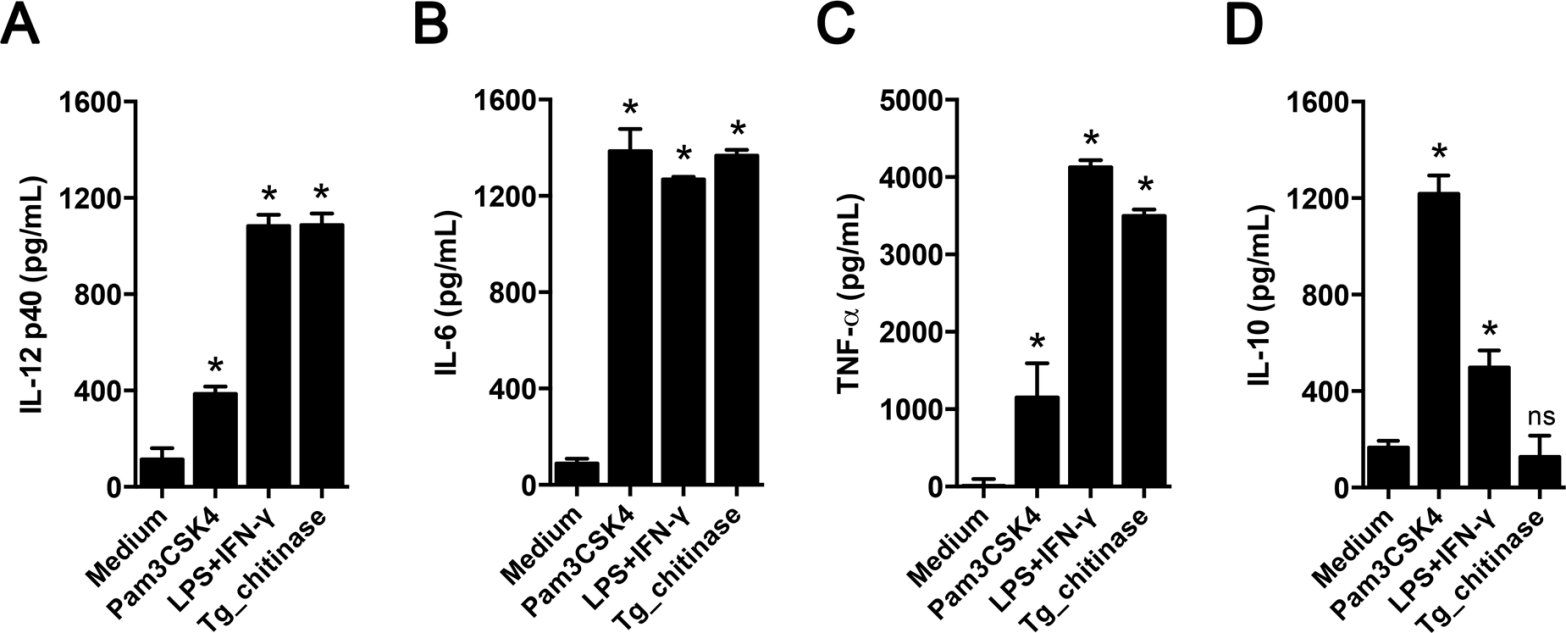
Tg_chitinase induces pro-inflammatory cytokines by peritoneal macrophages. Adherent cells from BALB/c mice peritoneal cavities were stimulated *in vitro* for 48 h with purified chitinase from *T*. *gondii*. Pam3CSK4 (1 μg/mL), LPS+IFN-γ (1 μg/mL and 1.5 ng/mL) were used as a positive controls. The culture supernatants were assayed for (A) IL-12, (B) IL-6, (C) TNF-α, and (D) IL-10 levels. Data is from a minimum of 3 independent experiments (mean ± SD). *p < 0.05 between Tg_chitinase and medium.

Considering that several chitinases and chitinase-like proteins play important roles in mediating and directing immune responses (reviewed by [10]), we investigated the effect exerted by Tg_chitinase on cytokine production by peritoneal macrophages. Cells were obtained from BALB/c and C57BL/6 mice, which are susceptible and resistant to *T*. *gondii* infection, respectively. A dose-response curve showed that chitinase concentrations of 1, 2.5, 5, and 10 μg/mL were effective in stimulating cytokine production. Following treatment with 2.5 μg/mL of Tg_ chitinase, murine macrophages were elicited from both BALB/c and C57BL/6 mice, increasing the production of IL-12, IL-6, and TNF-α, and reaching levels as high as the positive controls (i.e., Pam3CSK4 and LPS) (Fig 5A–5C). In contrast, Tg_chitinase did not induce peritoneal macrophages to produce the anti-inflammatory cytokine IL-10 (Fig 5D), as was observed in the positive controls.

## Discussion

We have demonstrated for the first time the isolation and partial characterization of a chitinase from *T*. *gondii*. The purified enzyme can be assigned to the class of β-acetyl-glucosaminidases based on its ability to cleave pNPGlcNAc. The adopted purification procedure, based on an affinity towards specific antibodies for paracoccin, a chitinase from *P*. *brasiliensis*, yielded a single 13.7 kDa protein, as verified by SDS-PAGE analysis. This protein is apparently responsible for NAGase activity and received the provisional name Tg_chitinase. The Michaelis constant (*Km*; 0.34 mM) indicated that this chitinase has higher affinity for the substrate than chitinases previously identified in *T*. *cruzi* (1.5 mM), *Clostridium paraputrificum* (7.9 mM), and *Trichoderma harzianum* (8.06 mM) (Table 1). Although few other chitinases from microorganisms have been described (Table 1), some of their characteristics were elucidated herein: (a) the optimal pH for chitinase activity is usually in the range of 4.0–7.0; (b) the optimal temperature is usually 50°C; and (c) their molecular mass is commonly between 15 and 50 kDa.

Our research on a chitinase from *Paracoccidioides brasiliensis* (i.e., paracoccin) identified an enzymatic domain and a carbohydrate-recognition domain (CRD) specific to GlcNAc [21, 22]. Our limited MS analyses of Tg_chitinase allowed for its identification as a protein derived from *T*. *gondii*, and also revealed a region with possible homology with a putative CRD from *B*. *subtilis* [28]. This suggests that Tg_chitinase and paracoccin have similar features. Nonetheless, additional studies are required to elucidate the domains of Tg_chitinase. Indeed, NAGase activity was previously detected in *T*. *gondii*-infected mammalian cells; the magnitude of enzymatic activity varied with infection duration [29]. Nevertheless, the study did not identify the enzyme implicated, or its origin, whether derived from the parasite or host cell. NAGase activity has also been detected in *T*. *cruzi* epimastigotes [30], but its biological role is unknown. Otherwise, chitinases from *Leishmania* spp. [12] and *Plasmodium* spp. [13] were reported to play important roles in disrupting chitinous structures of the digestive system of its invertebrate host, making transmission to the mammalian host possible.

It is plausible that Tg_chitinase has an integral role in *T*. *gondii* infection based on its similarity to a mammalian chitinase that controls the protozoan cyst burden in the brain. Despite the absence of GlcNAc polymers in vertebrates, chitinases and chitinase-like proteins (C/CLPs), which are hydrolytically inactive, are well conserved in vertebrate species. Several studies have shown that these proteins are involved in mediating the degradation of the chitinous exterior of pathogens, similar to what occurs in *T*. *gondii* cysts or in immune response regulation (Reviewed by [10]). Chitinases and CLPs are consistently reported as stimulating alternative macrophage activation, augmenting adaptative Th2 immunity, and mediating the effector functions of IL-13 [31]. Several authors consider that C/CLPs play a pivotal role in both innate and adaptive type 2 immunity. Intriguingly, the two chitinases derived from pathogens that had their immunomodulation properties investigated (i.e. paracoccin and Tg_chitinase), induced the production of Th1 cytokines by macrophages. We showed herein that Tg_chitinase activates macrophages from both C57BL/6 and BALB/c mice, augmenting levels of IL-12, IL-6, and TNF-α, but not IL-10. Paracoccin induced high production of Th1 cytokines and was able to confer protection against fungal infection in mice. A second Tg_chitinase sequence identified by MS was homologous with a peroxiredoxin PX3. This enzyme is known to induce an alternative pattern of macrophage activation associated with IL-10 production [32], therefore, we do not consider it as a component of *T*. *gondii* tachyzoites.

The results of the present study suggest that Tg_chitinase induces the production of Th1 cytokines, especially IL-12, which favors the hypothesis that this enzyme might influence acute phase pathogenesis of *T*. *gondii* infection. It is well established that cytokines are crucial in the development of toxoplasmosis. The production of pro-inflammatory cytokines, such as IL-12, are essential to develop a T helper 1 response, characterized by the presence of large amounts of interferon gamma (IFN-γ), which protects against infection [33]. Interferon gamma release triggers nitric oxide production by macrophages, promoting the intracellular destruction of protozoans, [34] and the differentiation of tachyzoites into bradyzoites, which replicate more slowly and form cysts in brain and muscle tissues [35]. Considering that enzymes are crucial for pathogenesis [36], and the fact that Tg_chitinase stimulates macrophages to release pro-inflammatory cytokines, especially IL-12, allows us to postulate that it might play an important role in the pathogenesis of toxoplasmosis, thereby contributing to the increase in slowly dividing bradyzoites that reside in tissue cysts.

## Materials and Methods

### Ethics Statement

This study was approved by the Ethics Committee and the Institutional Review Board of the University of São Paulo [approval number 065/2012].

### Toxoplasma gondii culture

We used the highly virulent RH strain of *T*. *gondii* in all experiments. The parasite was maintained by intraperitoneal passage in female Swiss mice (*Mus musculus*), aged 6–7 weeks, which were purchased from the animal house of the Campus of Ribeirao Preto, University of Sao Paulo. All mice were maintained in small groups inside isolator cages with light/dark cycle of 12 hours, besides food and water ad libitum were provided, in the animal housing facility of School of Medicine of Ribeirao Preto—Univesity of Sao Paulo. The mice were humanely euthanized by CO_2_ chamber and all efforts were made to minimize animal suffering. The parasites were collected 48–72 h after infection as previously described [37, 38]. Parasites obtained from peritoneal exudates were washed with phosphate-buffered saline [PBS; 10 mM sodium phosphate containing 0.15 M sodium chloride (both w/v) at pH 7.2] and centrifuged at 1000 ×*g* for 10 min. The pellet was then lyophilized and stored at -20°C until use.

### Soluble Toxoplasma gondii antigen (STAg)

The lyophilized pellet was re-suspended in PBS containing 0.8 mM phenylmethylsulfonyl fluoride (Sigma Chemical Co., St. Louis, MO, USA) and sonicated (Fisher Scientific, Pittsburgh, PA, USA) as described in [39], with three pulses at 10 kHz and 50 W for 50 s each. Soluble proteins were recovered by centrifugation at 3000 ×*g* for 30 min at 4°C and constituted the crude extract. The protein concentration was quantified by the colorimetric method of the bicinchoninic acid assay (BCA; Pierce Chemical Co., Rockford, IL, USA) using bovine serum albumin as the standard.

### Purification of N-acetyl-β-d-glucosaminidase (NAGase) from Toxoplasma gondii

NAGase was purified by immunoaffinity chromatography on an IgY-Sepharose 4B column equilibrated and incubated with slow rotation overnight at 4°C. The purified product was washed with PBS until the flow through had an OD280 less than 0.002. The bound protein was eluted with 0.1 M glycine at pH 2.5 and subsequently neutralized with 1 M tris-HCl at pH 8.5–9.0. The material was dialyzed against PBS using an Amicon® Pro Purification System (W. R. Grace & Co., Beverly, MA, USA) with a YM-10 membrane (Millipore Co., Billerica, MA, USA). The protein concentration was quantified as described above.

### Enzyme assay and characterization

NAGase activity was measured colorimetrically using the substrate pNPGlcNAc (Sigma) as previously described [40] with slight modifications [21, 22]. The total reaction volume (0.5 mL) contained 0.1 mL of 5 mM pNPGlcNAc (w/v), 0.35 mL of 0.1 M sodium acetate buffer (w/v), and 0.05 mL (50 μg) of crude extract. After incubation at 37°C, 1 mL of 0.5 M sodium carbonate (w/v) was added to the mixture to stop the reaction. The amount of liberated *p*-nitrophenol was measured spectrophotometrically at 405 nm. One unit of enzyme activity was defined as the amount of protein required to produce 1 μM *p*-nitrophenol per min at 37°C. The reported enzyme activity values correspond to the mean values of at least three replicates. In order to determine the *Km* value, we used a non-linear regression analysis of the data obtained by measuring the rate of pNPGlcNAc hydrolysis (from 0.01 to 0.1 mM; w/v), using GraphPad Prism v. 6.00 (GraphPad Software, San Diego, CA, USA). To determine the optimal pH and temperature for enzyme activity, enzymatic reactions were conducted using 0.1 M phosphate citrate buffer (pH 2.5–5.0; w/v) or 0.1 M sodium acetate buffer (pH 5.5–7.5; w/v) at a range of temperatures (25–65°C).

### SDS-PAGE analysis and protein identification by mass spectrometry

Sodium dodecyl sulfate-polyacrylamide gel electrophoresis (12% SDS-PAGE) was conducted according to [41]. Pre-stained standards (PageRuler; Fermentas, Ontario, Canada) were used to estimate the molecular weights of the sample proteins. The samples were stained with 0.25% Coomassie Brilliant Blue R-250 (w/v) and destained with a mixture of methanol/acetic acid/water (v/v/v; 4:1:5). After SDS-PAGE, the purified NAGase band was manually excised from the gel, prepared, and analyzed by LC-MS/MS mass spectrometry (XEVO-TQS mass spectrometer; Waters, San Diego, CA, USA) coupled with a UPLC chromatography system (Waters, San Diego, CA, USA) according to [42]. Raw data were converted to mzXML and automatically processed using Labkey Server v. 12, using theX!Tandem search algorithm [43].

### Immunofluorescence

For subcellular NAGase localization, we used tachyzoites of *T*. *gondii* RH strain, which were freshly isolated from infected mice using peritoneal washes. Parasites were fixed in 4% paraformaldehyde (w/v) at room temperature for 20 min, and then allowed to adhere to Biobond-treated coverslips for 15 min. Permeabilization was conducted with 0.3% Triton X-100 (w/v) for 20 min followed by a 30 min blocking period with 0.3% bovine serum albumin (BSA) diluted in PBS. A 1:100 dilution of anti-NAGase IgY-biotinylated antibodies was incubated with parasites at room temperature for 1 h. After five washes with PBS, the parasites were incubated with anti-streptavidin-FITC antibody for 30 min. All samples were then rinsed in PBS and coverslips were mounted with ProLong™ antifade reagent (Life Technologies, Carlsbad, CA, USA) and examined with a Leica TCS SP5 scanning confocal microscope (Carl Zeiss, Jena, Germany).

### Cytokine production analysis

Thioglycolate-elicited macrophages were stimulated with NAGase (0.5, 1, 2.5, 5 and 10 μg/mL) for 48 h in the cytokine production assay. Pam3CSK4 (1 μg/mL), LPS (1 μg/mL), and IFN-γ (1.5 ng/mL) (Invitrogen, San Diego, CA, USA) were used as positive controls. IL-6, IL-12p40, TNF-α, and IL-10 levels in the supernatant of peritoneal macrophage cultures from BALB/c and C57BL6 mice were measured by the capture enzyme-linked immunosorbent assay (ELISA) with antibody pairs purchased from BD Biosciences (Pharmingen, San Diego, CA, USA). The ELISA procedure was performed according to the manufacturer’s protocol. The quantity of cytokines was determined from a standard curve, using murine recombinants IL-12p40, IL-6, TNF-α, and IL-10.

### Prediction of three-dimensional (3D) structures and statistical analysis

Three-dimensional structures were predicted using I-TASSER [25], as previously described [44]. Data were analyzed using GraphPad Prism v. 6.00 (GraphPad Software, San Diego, CA, USA) and tested by one-way ANOVA followed by Bonferroni multiple comparisons. The data analyzed were the means from at least three independent experiments performed in triplicate. P values less than 0.05 were considered statistically significant.
